# Diseases Caused by *Xylella fastidiosa* in *Prunus* Genus: An Overview of the Research on an Increasingly Widespread Pathogen

**DOI:** 10.3389/fpls.2021.712452

**Published:** 2021-08-13

**Authors:** Davide Greco, Alessio Aprile, Luigi De Bellis, Andrea Luvisi

**Affiliations:** Department of Biological and Environmental Sciences and Technologies, University of Salento, Lecce, Italy

**Keywords:** *Xylella fastidiosa*, almond, peach, plum, cherry

## Abstract

Cultivated plants belonging to the genus *Prunus* are globally widespread and for some countries, are economically important crops; and they play a key role in the composition of a landscape. *Xylella fastidiosa* is a key threat to plant health, and several *Prunus* species are heavily stressed by this pathogen, such as almond, peach, and plum; many strain types of different subspecies can cause severe diseases. This review highlights different approaches to managing epidemic events related to *X. fastidiosa* in stone fruit plants. In fact, in most new European and Asian outbreaks, almond is the main and very common host and peach, plum, apricot, and cherry are widespread and profitable crops for the involved areas. Various diseases associated with stone fruit plants show different degrees of severity in relation to cultivar, although investigations are still limited. The development and selection of tolerant and resistant cultivars and the study of resistance mechanisms activated by the plant against *X. fastidiosa* infections seem to be the best way to find long-term solutions aimed at making affected areas recover. In addition, observations in orchards severely affected by the disease can be essential for collecting tolerant or resistant materials within the local germplasm. In areas where the bacterium is not yet present, a qualitative-quantitative study on entomofauna is also important for the timely identification of potential vectors and for developing effective control strategies.

## Introduction

*Xylella fastidiosa* is a gram-negative, xylem-limited, and slow-growing bacterium transmitted by some xylem-feeding vectors (Wells et al., 1987), and it is the causal agent of several plant diseases (Hopkins and Purcell, 2002). This plant pathogen is included in the EPPO A2 list of quarantine pathogens and is now present in many countries where it infects over 550 different species belonging to 80 different families (EFSA, 2020). *X. fastidiosa* is currently divided into three main subspecies, each with a specific host range: *X. fastidiosa* subsp. *fastidiosa* (which causes one of the most dangerous grapevine diseases called *Pierce's disease*, PD); *X. fastidiosa* subsp. *multiplex* (primarily associated with forest trees or *Prunus* spp.); *X*. fastidiosa subsp. pauca (well-known because of citrus variegated chlorosis, CVC, and olive quick decline syndrome, OQDS; Schaad et al., 2004; Martelli et al., 2016). Other subspecies are associated with diseases of less economic interest and with a limited host spectrum: *X. fastidiosa* subsp. *sandyi* (Schuenzel et al., 2005); *X. fastidiosa* subsp. *morus* (Nunney et al., 2014), and *X. fastidiosa* subsp. *tashke* (which causes leaf scorch in *Chitalpa tashkentensis*; Randall et al., 2009). Every subspecies is subdivided into sequence types (STs), each with different host ranges (Sicard et al., 2018; Nunney et al., 2019).

Some species belonging to the *Prunus* genus are among the most important hosts of different *X. fastidiosa* subspecies. In fact, almond leaf scorch disease (ALSD), phony peach disease (PPD), and plum leaf scald (PLS) are of considerable importance in the agricultural history of the United States and South America due to the damage they cause. Moreover, emerging *X. fastidiosa* outbreaks in Europe and Asia represent a significant threat for the *Prunus* cultivated for fruit production or ornamental purposes.

This review, thus, aims to generate a new interest in the research on diseases caused by *X. fastidiosa* in the *Prunus* species.

## Bibliographical Search

To evaluate the research involving diseases caused by *X. fastidiosa* on species belonging to the genus *Prunus*, an analysis of the bibliography of some of the most commonly used databases was carried out. This was done using StArt (State of the Art through Systematic Review) version 2.3.4.2 (http://lapes.dc.ufscar.br/tools/start_tool) developed by the Federal University of São Carlos (Brazil).

For this analysis, the databases used were Scopus (https://www.scopus.com), Springer (https://link.springer.com), PubMed (https://www.ncbi.nlm.nih.gov/pmc), and Google Scholar (https://scholar.google.com).

The literature search yielded 1,229 articles; however, only 128 of these were relevant. The results showed that most of the accepted articles (39%) were from the Scopus database, while the PubMed database contributed the least (18%); Springer and Google Scholar contributed 20 and 23%, respectively. The highest frequency was recorded in articles related to almond leaf scorch, while the lowest was for articles on *Xylella* in *Prunus* spp.

**Figure 1:**
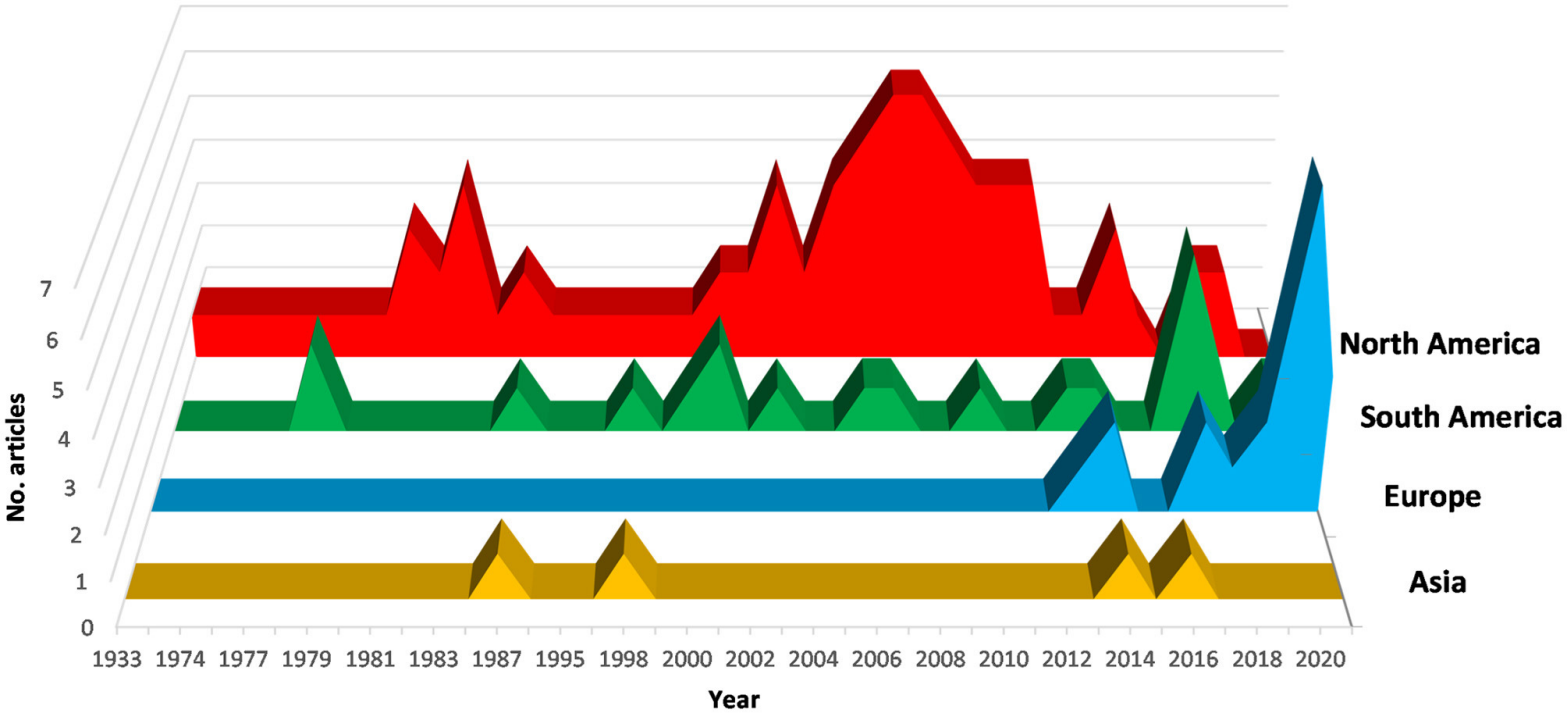
Trend from 1974 to 2021 in research on *X. fastidiosa* in plants belonging to the genus *Prunus*.

A total of 75% of the articles were published between 2000 and 2021, 24.4% between 1974 and 1999 (Figure 1). North and South America have contributed most to *X. fastidiosa* research on stone fruit, accounting for 82.7% of the total number of published studies, with a peak from 2004 to 2012. In these areas, the bacterium has been studied for a long time, and resulting diseases have had historical significance.

In Europe, after the first detection of *X. fastidiosa* in 2013, the scientific research increased considerably. In particular, Spain and Italy, which are the most affected by the new epidemics, are strongly involved in research on *X*. *fastidiosa* in *Prunus* spp. After 2013, these two countries accounted for 45% (30% Spain; 15% Italy) of the total number of scientific articles, much more than North and South America each contributing 25% of the published articles.

**Figure 2:**
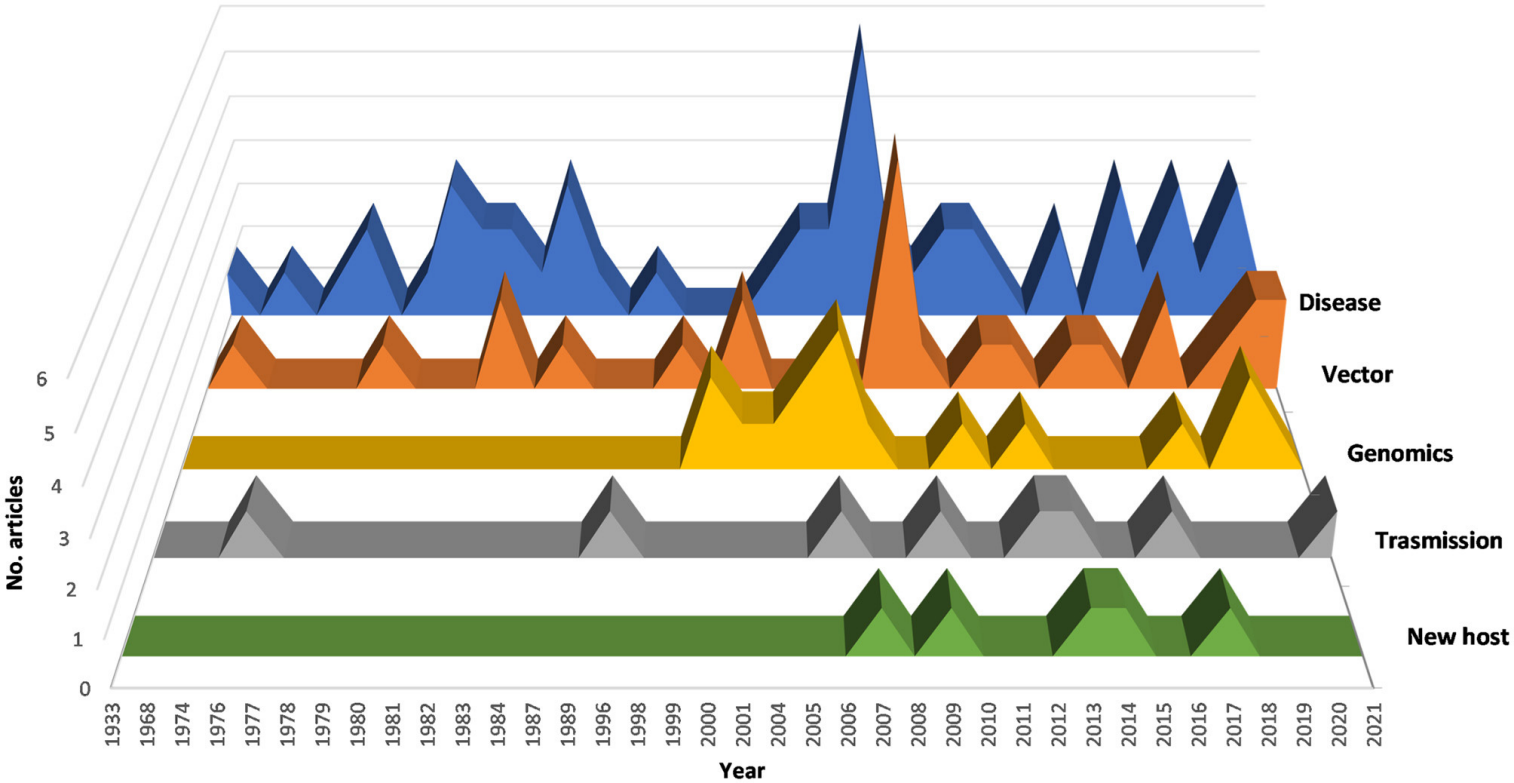
Trend from 1974 to 2021 on the research topics analyzed.

The most frequent research topics (Figure 2) are those relating to the category “disease” (48.1% of the total number of articles), and include symptoms, epidemiology, diffusion, and diagnosis of diseases. Vectors (biology and diffusion) were studied in 23.6%, while bacterium transmission (*via* insect or graft) was in 7.6% of the studies.

From 2000 onward, articles on the genomic study of different subspecies of the bacterium represented 20.3% of the total. In fact, the complete sequencing of the *X. fastidiosa* genome took place in 2000 (Simpson et al., 2000). The recent discovery of *X. fastidiosa* in Spain, Italy, and Iran, countries which are some of the most important stone fruit producers, alerted the agricultural sector and the scientific community, which immediately began to study the phenomenon. In these countries, the interest of scientists in better understanding this bacterium derives from the American experience of diseases caused by *X. fastidiosa*, and from the closest Apulian experience where OQDS destroyed many olive orchards.

## History and Spread of Diseases Caused by *Xylella fastidiosa* in *Prunus* Species

### Almond Tree

The almond (*Prunus dulcis* L.) is native to Central Asia and, with Arab invasions in the tenth century, was introduced into all Mediterranean basin countries. In the sixteenth century, through the Spanish colonization of the Americas, the almond tree was imported to the United States (Losciale, 2016), which is currently the largest almond producer globally (www.fao.org/faostat/en/, FAO Code: 0221-Almonds, with shell).

The almond tree has several biotic adversities; however *X. Fastidiosa* causes the most damage, especially in California (CA, United States), where almond-growing is widespread. The first observation linked to ALSD dates to 1930 in Riverside Country (CA) (Mircetich et al., 1976; Almeida and Purcell, 2003a). This disease was described in 1974 (Moller et al., 1974) and was known as the “golden death.” Years later, in 1994, on a roadside in San Joaquin Valley (CA), Sandy Purcell found almond trees with leaf scorch symptoms typically associated with *X. fastidiosa* infection, thus establishing the first hypothesis of the connection between this pathogen and the symptoms observed. Shortly after, two almond trees (cultivar “Sonora”) were found to be infected in Kern Valley (CA) (Haviland and Viveros, 2005). In 2003, ALSD emerged again as a severe threat to almond production in San Joaquin Valley (California) (Almeida and Purcell, 2003a).

In Europe, monitoring began after the discovery of the first outbreak of *X. fastidiosa* in the Salento peninsula (Apulia, Italy) in 2013 (Saponari et al., 2013). Here, thousands of olive trees were affected by a severe desiccation syndrome, the OQDS (Martelli et al., 2016). Together with olive trees, almond trees were found to be infected and symptomatic, with typical leaf scorch symptoms of ALSD (Saponari et al., 2013), but with low levels of infection compared with infected olive trees (Saponari et al., 2017). Shortly afterward, in another area of Italy, *X. fastidiosa* was discovered in the Monte Argentario promontory in Tuscany, while official monitoring of the pathogen was being performed. In this outbreak, an almond tree was also found among the infected plants (Marchi et al., 2018).

A further European case of particular importance was the ALSD outbreak in Majorca, the largest island in the Balearic Islands (Spain), where the first report of *X. fastidiosa* dates to 2016 in a cherry tree (Olmo et al., 2017). Subsequently *X. fastidiosa* was also found in many symptomatic almond trees throughout the island (Moralejo et al., 2020). For Majorca, almond trees are symbolic of the rural landscape; and over the past 15 years, in many almond orchards a severe decline syndrome has been observed (Gramaje et al., 2012). At first, this was associated with a complex of fungal trunk pathogens (Olmo et al., 2015, 2016) before the detection of *X. fastidiosa*. The bacterium, thus, seems to have been introduced in the 1990s, when American almond cultivars were grafted onto local almond rootstocks in two orchards, one of which is located in Son Carrió, where the first diseased almond trees were observed (Gramaje et al., 2012; Moralejo et al., 2020).

Subsequent monitoring also found *X. fastidiosa* on other islands of the Balearic archipelago, such as Ibiza and Minorca (Olmo et al., 2021). Almond trees infected by *X. fastidiosa* have also been detected in the Iberian Peninsula, in the municipality of El Castell de Guadalest (Alicante province, Spain). Later, during an official survey carried out in the spring of 2018, the infection was found in more than 170 almond orchards in 27 different municipalities (Jeger et al., 2018). The bacterium was not only detected in almond trees but also in other *Prunus* species, such as plum and apricot trees (Ferrer et al., 2019). In 2015, France announced the detection of *X. fastidiosa* in almond trees in Corsica. The number of infected plants rapidly increased until Commission Implementing Decision (EU) 2017/2352 declared the entire island under containment.

Outside Europe, this quarantine-listed pathogen has been detected in Iran and Israel (EFSA, 2020). In Iran, *X. fastidiosa* was detected in the summer 2014 in many almond orchards and vineyards with some symptomatic plants. ALSD was also confirmed in commercial almond orchards in Chahar Mahal-va-Bakh-tiari, West Azerbaijan and Semnan provinces (Amanifar et al., 2014). At the same time, in the Razavi-Khorassan province, a severe apricot decline syndrome was observed, which had been previously associated with phytoplasmas, but some plants also tested positive for *X. Fastidiosa* (Karimishahri et al., 2016). In Israel, during a general inspection throughout the country from 2017 to 2018, almond trees were found to be affected by *X. fastidiosa* in three commercial orchards in the Hula Valley (EPPO, 2019).

### Plum and Peach Trees

Two plum species dominate modern world production: European plum (*Prunus domestica*) and Japanese plum (*Prunus salicina* Lindl.). The European plum originated in the southern region of the Caucasus mountains, whereas the Japanese plum originated in the Yangtze River basin (China). A different historical diffusion made the European plum dominant in Europe and the Japanese plum dominant elsewhere (Topp et al., 2012).

After China, the United States is the second largest producer of plums in the world with an average of 538,483 tons between 1994 and 2019 (www.fao.org). In the USA, California is the main producer (Topp et al., 2012) of the Japanese plum with 25.4% and the European plum with 74.6% (USDA, 2020).

Peaches are one of the most important temperate fruit species cultivated globally, and all the cultivars belong to the *Prunus persica* (L.) Batsch species (Byrne et al., 2012). This species has Asian origins and was domesticated in China, from where it reached all the continents (Byrne et al., 2012). The United States is the fourth leading producer of peaches after China, Italy, and Spain, with an average output of 1,169,709 tons between 1994 and 2019 (www.fao.org).

Plums and peaches are also both important hosts of *X. fastidiosa*; however, PPD and PLS are present only in the Americas. PLS is a harmful disease of both Japanese and European plums in many South American countries (French and Kitajima, 1978; Chang and Yonce, 1987). This disease was first reported in 1935 in the delta region of the Paranà River (Argentina) (Fernandez-Valiela and Bakaracic, 1954; Chang and Yonce, 1987), and subsequently, the disease appeared in Brazil and Paraguay (French and Kitajima, 1978). In southern United States, symptoms of PLS were first observed in 1970; however, first official reports were issued some years later (French and Kitajima, 1978).

PPD was first observed toward the end of the nineteenth century in Georgia (United States) in the vicinity of the town of Marshallville (Neal, 1920). Over the years, the disease reached six counties of Georgia (Overall and Rebek, 2017). Once the danger of PPD was understood, monitoring was carried out between 1929 and 1952. A survey led to the detection of PPD in Alabama, northern Florida, Louisiana, Mississippi, South Carolina, southern Arkansas, and eastern Texas (Turner and Pollard, 1959). Between 1929 and 1947, PPD led to the loss of about 1,500,000 peach trees (Turner, 1949). To date, in the United States, PLS and PPD have caused an enormous economic damage, mainly in orchards in Florida and Georgia (Dutcher et al., 2005; Overall and Rebek, 2017).

## Symptoms, Subspecies, and Sequence Types

### Almond Leaf Scorch Disease

ALSD has a gradual onset. Characteristic symptoms are marginal leaf scorch, which is observed in late summer, when the temperature and water demand increase (Teviotdale and Connell, 2003). The first symptoms occur only on some branches, after which, in subsequent years, the plant becomes seriously ill, showing a typical golden-brown appearance (“*golden death*”; Sanborn et al., 1974). ALSD can be distinguished from leaf symptoms caused by salt stress by the presence of a yellow band on the leaf tissue between the green and scorched areas (Sanborn et al., 1974). The latency period of this disease seems to be variable, depending on the inoculation season; almond trees infected in the spring can develop more severe symptoms in comparison with plants infected in the summer and autumn (Cao et al., 2011).

According to some studies, the first symptoms appear 8–10 weeks after inoculation (Ledbetter and Rogers, 2009; Cao et al., 2011). On the other hand, Davis et al. (1980) observed that almond trees inoculated in the spring (March) and summer (June) showed symptoms 3 months later. More recently, Marco-Noales et al. (2021) observed disease symptoms a year after inoculation.

In areas affected by ALSD, especially in California, observations over the years have underscored different degrees of susceptibility among cultivars (Moller et al., 1974; Sanborn et al., 1974). The incidence seems to be most severe in the cultivars “Jordanolo,” “Long IXL,” “Mission,” “Ne plus ultra,” “Nompareil,” “Peerless,” “Price,” “Solano,” “Sonora,” and “IXL.” “Sonora” seems to be the most susceptible. The cultivars “Butte” and “Carmel” are considered as resistant (Moller et al., 1974; Sanborn et al., 1974; Cao et al., 2011; Wilhelm et al., 2011). Ten of the most susceptible cultivars were found to represent 86% of those grown in California (Mircetich et al., 1976).

For a long time, it was thought that ALSD and PD were caused by the same *X. fastidiosa* strain. Later however, it was observed that under greenhouse conditions, all isolates from grapes and almonds caused ALSD, while some isolates from almonds did not cause PD (Almeida and Purcell, 2003a). These different strains were isolated from almond trees located in Kern Valley (CA, United States) and were called M12 and M23. Subsequently, the first isolated strain was named the A-genotype (almond genotype) because it only caused ALSD; the second was named G-genotype (grape genotype), which caused both PD and ALSD (Chen et al., 2005).

The A-genotype corresponds to subsp. *Multiplex*, and the G-genotype corresponds to subsp. *fastidiosa* (Schaad et al., 2004). These subspecies act differently on almond trees. Subsp. *fastidiosa* reaches a bacterial concentration 10-fold lower in almonds than in grapes; however, the incidence of ALSD and its severity are greater than that recorded for subsp. *multiplex* (Chen et al., 2005; Groves et al., 2005). Mircetich et al. (1976) observed that the colonization rates of xylem vessels in affected almonds were considerably lower (10–15%) in comparison with infected grapes, where nearly 20% of vessels were occluded. Almeida and Purcell (2003b) recorded a variable bacterial density in leaf petioles taken from almond trees affected by *X. Fastidiosa*, which ranged between 10^5^ and 10^7^ cfu/g of tissue. Instead, in grapes affected by PD, the bacterial density ranged between 10^8^ and 10^9^ cfu/g of tissue. The *X. fastidiosa* strains inoculated during this study were strain Tulare for almonds and strain STL for grapes, which both belong to the subsp. *fastidiosa* (Hernandez-Martinez et al., 2006). No data are available regarding the cultivars evaluated (Almeida and Purcell, 2003b).

ALSD strains belonging to *X. fastidiosa* subsp. *multiplex* were subdivided into ALSI and ALSII, two different groups distinguished by random amplification of polymorphic DNA (RAPD) analysis (Almeida and Purcell, 2003b). ALSD appears to be caused by several sequence types (STs) belonging to several *X. fastidiosa* subspecies, such as *fastidiosa, multiplex, pauca*, and *sandyi*. *X. fastidiosa* STs causing ALSD are shown in Table 1. They were classified by multi locus sequence typing (MLST), which is widely performed in molecular biology (Maiden et al., 1998) to recognize and regroup bacterial genotypes based on sequences of seven housekeeping genes.

**Table 1:** Sequence types found in almond trees (*P. dulcis*) and location (Jeger et al., 2018; Amanifar et al., 2019; Bahar et al., 2019; EPPO, 2019; Saponari et al., 2019; EFSA, 2020; www.pubmlst.org).

**Subspecies**	**Sequence type**	**Location**
*Fastidiosa*	ST1 (ALSI, Tulare, M23, G-genotype, STL)	USA (California), Spain (Majorca), Israel (Hula Valley)
*Multiplex*	ST6 (ALSII)	USA (San Joaquin County-California), Spain (Majorca, Alicante), France (Corsica)
	ST7 (M12, A-genotype)	USA (Kern County-California), Spain (Majorca), France (Corsica)
	ST27	USA
	ST81	Spain (Majorca), Spain (Menorca)
	ST87	Italy (Tuscany)
	-	Iran (Chahar Mahal-va-Bakh-tiari, West Azerbaijan, Semnan)
*Pauca*	ST53	Italy (Apulia), France (Corsica)
	ST80	Spain (Ibiza)
	ST78	Argentina

According to Amanifar et al. (2019), in Iran, there are two subspecies of this plant pathogen after considering gene sequencing and differences in biological and morphological traits of bacterial colonies, namely, subsp. *fastidiosa* isolated from grapes and subsp. *multiplex* isolated from pistachios and almonds (Amanifar et al., 2014, 2016). However, more gene sequencing is necessary to determine the sequence types present in Iran.

Two different subspecies can exist simultaneously and cause ALSD in the same orchard. The first case reported is a study by Chen et al. (2005), which described the coexistence of subsp. *fastidiosa* and subsp. *multiplex* in two ALSD-affected orchards located in Fresno County (San Joaquin Valley, California). Another case is a phytosanitary emergency in Majorca, where ALSD was caused by the subspecies *fastidiosa* ST1 and *multiplex* ST7/ST81. Here, both the subspecies were detected in the same orchard and even within the same plant (Moralejo et al., 2020).

The incidence and severity of ALSD depend on several factors, such as orchard location, cultivar, management of irrigation, and nutrition (Amanifar et al., 2016). Studies in the United States have reported an incidence in the field ranging from 0.08 to 17% (Sisterson et al., 2008, 2012; Ledbetter and Rogers, 2009; Cao et al., 2011; Daane et al., 2011; Wilhelm et al., 2011). In Majorca, the incidence was between 16.6 and 100%, with an average incidence of 79.5% (Moralejo et al., 2020). In France *X. fastidiosa* subsp. *pauca* ST53, subsp. *multiplex* ST6 and ST7 have also been recorded in Provence-Alpes-Côte d'Azur (PACA) and Corsica; however, they were not detected in *Prunus* spp. (Jeger et al., 2018). Sisterson et al. (2008) reported a reduced production by 20–40%, and the yield losses of infected trees did not increase year after year with very low mortality (about 9%) (Sisterson et al., 2008, 2012). However, other studies have shown how infected orchards consistently reduced their yield every year with ALSD-affected trees dying 3–8 years after disease onset (Sanborn et al., 1974; Mircetich et al., 1976; Teviotdale and Connell, 2003; Haviland and Viveros, 2005).

### Phony Peach Disease, Plum Leaf Scald, and Other Diseases

PPD is manifested by slender and shortened young twigs, with shorter than average internodes and more lateral ramifications, and with horizontal growth; the leaves are dark green and denser than normal. Budding, flowering, and fruit ripening occur early, whereas autumnal phyllotopsis occurs late. Initial symptoms can be observed in the whole canopy or only within a branch (Turner and Pollard, 1959; Mizell et al., 2008; Janse and Obradovic, 2010). No leaf scorching occurs in diseased peach trees (Mizell et al., 2008). Three to 5 years after the appearance of the first symptoms, production begins to decrease, and fruits become smaller and unsuitable for the market, with more intense coloring than fruits produced by healthy peach trees. A PPD-affected tree shows an umbrella-like canopy, which is less thick and less dense than an unaffected plant (Hutchins, 1933). In other *Prunus* spp., similar symptoms have been described, such as in wild plums (*Periplaneta americana* L.), apricots (*Prunus armeniaca* L.), and purple-leafed plums (*Prunus cerasifera* Ehrh.) (Turner and Pollard, 1959).

The latency time for this disease is ~18 months after inoculation (Janse and Obradovic, 2010). Symptoms appear in warm months, but during extremely dry summers, disease onset is later than usual (Janse and Obradovic, 2010). High winter temperatures promote many vectors that can cause new epidemics (Chen et al., 2019). Affected plants generally do not die but become more sensitive to biotic and abiotic factors (Janse and Obradovic, 2010). According to some studies, in PPD-infected peach trees, *X. fastidiosa* is present in higher concentrations in the xylem fluid of the root system (Aldrich et al., 1992; Chen et al., 2019). Consequently, only root samples allow reliable and stable detection throughout the year (Chen et al., 2019). PPD has not been detected in all states in the United States; and in South America, it appears to be a phytosanitary problem. The incidence of PPD is high near the Gulf of Mexico and low in south midwestern United States. In Georgia and North and South Carolina, the incidence of the disease decreases with increasing altitude above sea level (Hopkins and Purcell, 2002).

The development of rootstocks with the most sought-after features is fundamental for optimizing almond production in terms of quality and quantity, and peaches propagated by seeds are widely used in California as rootstocks for almond trees. Peach grafting provides insufficient vigor to almond trees, and some cultivars, such as “Nemaguard” or Okinawa, are resistant to nematodes *Meloidogyne incognita* and *Meloidogyne javanica* (Gillen and Bliss, 2005; Ledbetter and Rogers, 2009). When almond trees need more vigor, peach × almond hybrids are also used (Ledbetter and Rogers, 2015), while other *Prunus* species are used to develop hybrids. Ledbetter and Rogers (2009) showed that peach ×almond hybrids were not a good host for *X. fastidiosa* subsp. *fastidiosa* strain M23 (ST1). Ledbetter and Rogers (2015) also indicated that *Prunus webbii* (a wild almond) crossed with *P. persica* cv “Harrow Blood” (resistant to *X. fastidiosa*) produces a susceptible host for both subsp. *fastidiosa* and subsp. *multiplex*., representing an inoculum reservoir for new ALSD epidemics.

PLS exhibits leaf-scorching symptoms similar to those of PPD (Overall and Rebek, 2017). Disease progresses gradually; first striking the young shoots, and then entire branches. The infected plum trees are fully compromised in two or more years. The initial foliar symptoms are chlorosis of leaf margins, with subsequent formation of necrotic bands (Latham and Norton, 1980) and a gray or dark brown color, giving the plant a burnt look (scald) (Ducroquet et al., 2001). Before phillotopsis occurs, necrosis can involve more than half of the leaf blade. Diseased plants shed their leaves prematurely in September and October, and develop new malformed and leathery leaves (Latham and Norton, 1980).

Brazil is one of the countries affected by PLS the most. Between the 1970s and 1980s, there was a considerable reduction in yield and an increase in eradicating orchards in Minas Gerais, Rio Grande do Sul, and Paranà (Ferreira et al., 2016). PLS dramatically reduces the quality of plums, which have lower weight and diameter; fruits are also more sensitive to *Monilina fructicola* (Kleina et al., 2018). In Brazil, PLS was observed on 31 cultivars of *P. salicina* and six cultivars of *P. domestica*^1^. The most sensitive cultivar is “Santa Rosa,” representing 90% of cultivars in Brazil (French and Feliciano, 1982).

Latham et al. (1980) found more leaf scorching symptoms on some cultivars and on hybrids developed from species *P. americana, P. cerasifera, Prunus munsoniana, P. salicina, P. silochorus simoni*, and *Polygala triflora* compared with cultivars “Homeside,” “Mariposa,” “Morris,” and “Methley A-21,” which showed few symptoms in Alabama (United States).

*Xylella fastidiosa* subsp. *multiplex* causes PPD and PLS (Schaad et al., 2004), but other subspecies were also found in *Prunus* hosts. Table 2 lists all the sequence types detected on peach and plumtrees in countries where the presence of the pathogen has been officially identified.

**Table 2:** Sequence types found on peach (*P. persica*), European plum (*P. domestica*), and Japanese plum (*P. salicina*) trees and location (Della Coletta-Filho et al., 2017; EFSA, 2020; www.pubmlst.org).

**Host**	**Subspecies**	**Sequence type**	**Location**
Peach	*Multiplex*	ST26	USA (Riverside County-California)
		ST10	USA (Georgia, Florida, Orange County-California)
	*Pauca*	ST53	France (Corsica)
European plum	*Multiplex*	ST6	Spain (Majorca)
		ST10	USA (Georgia)
		ST26	USA (Riverside County-California)
		ST63	Brazil
		ST81	Spain (Majorca, Menorca)
	*Paucapauca*	ST71	Brazil
Hybrid plum	*Multiplex*	ST41	USA (Georgia)

Peach and plum trees can be infected by subsp. *pauca*, while subsp. *multiplex* is the etiological factor of PPD and PLS in countries where these diseases have long represented a serious phytosanitary problem.

Table 3 lists the sequence types found in other species of the *Prunus* genus. Of these, an uncommon disease caused by *X. fastidiosa* is leaf scorch of purple-leafed plum (*P. cerasifera*), which was observed during a survey conducted between 2003 and 2004 in southern California (United States). Purple-leafed plum is a small tree with fleshy purple leaves and pink flowers, and it is used as an ornamental plant all over the world. The symptoms of this disease are leaf scorching and plant decline, similar to those of ALSD and PLS (Hernandez-Martinez et al., 2009).

**Table 3:** Sequence types found in other *Prunus* species and locations (EFSA, 2020; www.pubmlst.org).

**Host**	**Subspecies**	**Sequence type**	**Location**
Apricot (*P. armeniaca*)	*Multiplex*	ST6	Spain (Alicante)
		ST46	USA (Riverside County-California)
		ST26	USA (Riverside County-California)
Purple leaf plum (*P. cerasifera*)	*Multiplex*	ST6	France (Corsica)
		ST7	France (Corsica)
		ST15	USA (Riverside County-California)
		ST34	USA (Riverside County-California)
		ST40	USA
Cherry (*P. avium*)	*Fastidiosa*	ST1	USA (San Bernardino-California), Spain (Majorca)
	*Pauca*	ST53	Italy (Apulia)
*Prunus* sp. (decorative prunus)	*Multiplex*	ST26	USA (Riverside County-California)

## Search for Resistant or Tolerant Cultivars

Selection of tolerant and resistant cultivars and understanding plant resistance mechanisms to counteract *X. fastidiosa* infections were originally the methods focused on to combat the bacterium. For example, crossbreeding and selection programs of new varieties have been established to safeguard the citrus sector from the advancement of CVC (Della Coletta-Filho et al., 2020), PD (Ramming et al., 2009), and OQDS (Boscia et al., 2017). Even genetically engineered plants may be a valuable solution to these diseases (Lindow et al., 2014; Caserta et al., 2017).

During the experience in California (United States) of ALSD on almond trees, field observations identified resistant or tolerant cultivars (Moller et al., 1974; Sanborn et al., 1974). As previously mentioned, the cultivars “Butte” and “Carmel” are considered as resistant because they reduce bacterial load in the winter months and usually show few symptoms and survive in fields heavily attacked by ALSD (Wilhelm et al., 2011). The disease was also reported to be less common in cultivars “Mission,” “Aldrich,” and “Padre” (Groves et al., 2005; Haviland and Viveros, 2005; Cao et al., 2011). According to Cao et al. (2011), the two main factors on which the occurrence of ALSD and its severity depend on the inoculation date and cultivar susceptibility. However, other factors such as winter temperatures and the duration of the cold period can influence the occurrence of the disease. In fact, the inoculation of 10 almond cultivars in the summer caused symptoms after about 2 months, but not all plants were diseased a year later. This means that the bacterium did not overwinter in a sufficient quantity to cause disease.

Wintercuring (or cold curing) has already been studied on grapes (Lieth et al., 2011) and almonds, and has been observed mostly in ALSD-resistant cultivars. Wilhelm et al. (2011) analyzed xylem sap year-around in susceptible and resistant almond cultivars in California to better understand the resistance mechanisms of almond trees against *X. fastidiosa*. Their results revealed that malic and citric acids were the major organic compounds in the almond xylem fluid, and that there were no differences in the composition of xylem sap among the tested cultivars. However, during the winter months, an increase in total phenolic compounds was observed in resistant cultivars (“Butte” and “Carmel”). In January, cultivar “Carmel” had a significant quantity of gallic acid, which is a phenolic compound already studied for its inhibitory activity against *X. Fastidiosa* in xylem sap (Maddox et al., 2010); in fact, secondary metabolites may play a central role in plant resistance to *X. Fastidiosa* (Ribeiro et al., 2008; Wallis and Chen, 2012; Luvisi et al., 2017).

The microbiome has also been studied to evaluate its possible resistance role in ALSD-affected and unaffected almond trees in Alicante (Spain) by Costa et al. (2019). These authors observed 77 operational taxonomic units (OTUs) that were present in both healthy and diseased plants; 32 and 38 OTUs were characteristic in healthy and diseased plants, respectively. Also, in olive trees, significant differences in microbiome composition between resistant and sensitive cultivars were observed by Vergine et al. (2020).

Rootstocks are widely used in *Prunus* spp., as they induce higher or lower vigor, and they also tolerate and resist various biotic or abiotic stresses. Krugner and Ledbetter (2016) observed a reduction in ALSD symptoms and complete recovery from the disease in almond trees grafted on peach “Nemaguard,” while almond trees grafted on “Okinawa,” “Nonpareil,” and “Y119” showed disease persistence year after year. In contrast, Cao et al. (2011) reported that rootstocks did not confer tolerance or, if there was, the contribution of rootstocks to symptom reduction was very low.

In Brazil, where Japanese plum is widespread, the adoption of resistant or tolerant plants appears to be able to reduce the economic impact of PLS and restore the plum production sector. Breeding programs started in the 1990s, crossing cultivars from Florida (United States) and the delta of the Parana River (Argentina) with susceptible local cultivars (Dalbó et al., 2010, 2016, 2018). Unfortunately, initial tolerant cultivars, such as “Carazinho,” “Sanguinea,” “Chatard,” and “Piamontesa,” had a very low-quality production (Dalbó et al., 2016). However, the selection program created a cultivar with exceptional characteristics, i.e., “SCS438 Zafira,” which, besides being resistant to PLS, also produces high-quality fruits (Dalbó et al., 2018). Its tolerance appears to be due to the influence of the plant-on the-insect behavior of the vectors.

Kleina et al. (2020) observed that two common vectors in Brazilian plum orchards, *Bucephalogonia xanthophis* and *Sibovia sagata*, prefer to feed on local susceptible cultivars, perhaps because of the presence of repellent substances in resistant genotypes (Dalbó et al., 2018). PLS-tolerant Japanese plum cultivars, suitable for both northern and southern plum orchards in Florida (United States), have also been bred, namely, “Gulfruby,” “Gulfbeauty,” “Gulfblaze,” and “Glulfrose” (Sherman and Rouse, 2001). These cultivars are also tolerant to *Xanthomonas campestris* pv. *Pruni*.

## Insect Vectors of *Xylella fastidiosa* on *Prunus* SPP. Around the World

*Xylella fastidiosa* is only able to colonize two substrates in nature: the xylem of host plants and the foregut of vectors. This bacterium is transmitted by several insects that feed on xylem sap (xylem sap-feeding insects), all belonging to the Hemiptera order, superfamilies *Cicadoidea, Cercopidea*, and *Cicadelloidea*. The subfamily of *Cicadellinae*, which are xylem-feeding insects, are vectors (Bosco, 2014). Vectors acquire the pathogen during feeding. In the insect body, the bacterium is not systemic and multiplies only in the upper part of the digestive system (Janse and Obradovic, 2010; Bosco, 2014). The rod-shaped bacteria attach themselves to the cuticle of the foregut in a polar orientation, apparently to better absorb nutrients and resist the turbulence of the xylem sap during the insect feeding (Overall and Rebek, 2017). The foregut has an ectodermal origin, and is renewed with the molt; thus, there is no transstadial transmission of the bacterium; moreover, the pathogen is not transmissible to the insect offspring (Almeida et al., 2005). After having accidentally acquired the bacterium, the adult vector insect can transmit it for the rest of its life (Mizell et al., 2008). Not many bacterial cells are required to cause disease in a healthy plant (Hill and Purcell, 1995), and the vector can transmit it immediately upon acquisition from an infected plant (Mizell et al., 2008). In a study carried out on *Graphocehala atropunctata*, the maximum bacterial level in the insect was reached seven days after ingestion (Hill and Purcell, 1995).

The vectors of *X. fastidiosa* belong to many species and differ from country to country. In North America, the most important vectors in almond orchards are *Draeculacephala minerva* (or green sharpshooters; Purcell, 1981; Janse and Obradovic, 2010) and *G. atropunctata* (or blue-green sharpshooter; Purcell, 1981; Redak et al., 2004). Other species that can transmit the bacterium are *Gymnopus confluens, Carneocephala fulgida*, and *Philaenus spumarius* (insects of European origin together with *Cicadella viridis*; Purcell, 1980; Janse and Obradovic, 2010). *D. minerva* was reported as the most common vector in Sacramento Valley (California), in and around almond orchards (Daane et al., 2011), and it has been found in large numbers in irrigated pastures and weedy alfalfa fields (Purcell and Frazier, 1985; Sisterson et al., 2008; Daane et al., 2011). Consequently, it is advisable to keep almond groves away from irrigated pastures or fields of alfalfa, because they are sources of vectors (Krugner et al., 2012). *G. atropunctata* is one of the most studied vectors and is characterized by a transmission efficiency of 100% in almonds and grapes (Redak et al., 2004).

The situation of *X. fastidiosa* vectors in the USA changed dramatically in the 1990s because of the accidental introduction of *Homalodisca vitripennis* (*H. coagulata* or glassy winged sharpshooter) from Mexico, which caused a sharp increase in *X. fastidiosa* disease outbreaks. In its area of origin (northern Mexico), this insect transmits PPD and PLS (Matthew et al., 2000), and it was probably introduced in the USA with nursery materials, as this insect lays its eggs in the woody tissues of plants (Janse and Obradovic, 2010). *H. vitripennis* is a threat for many crops because of the huge number of its host plants and its ability to travel a long distance in a short time, e.g., 180 m in 2 h (Redak et al., 2004; Mizell et al., 2008). Although its transmission efficiency is low compared with *G. atropunctata, H. vitripennis* can also infect grapes and almonds during winter dormancy (Almeida and Purcell, 2003b). *H. vitripennis* is currently widespread in south-eastern United States and northern Mexico (Redak et al., 2004), where it represents the main *X. fastidiosa* vector.

In southern United States and northern Mexico, the vectors identified for PPD include *H. vitripennis* and *H. insolita*, whereas other vectors are found southeast of the United States (*Oncometopia orbona* and *Cuerna costalis*). In South America, common vectors are *O. nigricans* and *G. versuta* (Redak et al., 2004; Overall and Rebek, 2017); whereas in Brazil, two widespread vectors in plum orchards are *Bucephalogonia xanthophis* and *Sibovia sagata* (Dalbó et al., 2018). The glassy-winged sharpshooter is the most important vector of PPD and PLS; however, peach also seems to be an occasional host. On sensitive plum cultivars “Methley” and “Santa Rosa,” many specimens of this insect are found during the summer (Andersen et al., 2008). A study showed that spraying imidacloprid onto the roots reduced the symptoms of PPD and PLS (Dutcher et al., 2005). In fact, imidacloprid is a systemic insecticide against *H. vitripennis* and many homopteran pests.

No American vectors of *X. fastidiosa* have been introduced in Europe yet (Olmo et al., 2021). Here, only few insects have been confirmed as being able to transmit these bacteria. However, in some cases, they seem to have played a crucial role in the spread of diseases, such as *P. spumarius* in Apulia, an area in Italy intensely affected by the OQDS epidemic. In Apulia, bacterial transmission was demonstrated for *P. spumarius, Neophilaenus campestris*, and *P. italosignus* (Cavalieri et al., 2019). For *P. spumarius*, it was also noted that bacterial acquisition from almond trees was low compared with the olive tree (Cornara et al., 2017). As for cherry trees, only *P. italosignus* seems to transmit the bacteria (Cavalieri et al., 2019). This could explain the low number of infected almond and cherry trees in the infected area of the Salento peninsula compared with olive trees (Cavalieri et al., 2019). In the promontory of Argentario (Tuscany, Italy), no vector has yet been confirmed, but a large *P. italosignus* population has been detected (Panzavolta et al., 2019).

In Majorca, both subspecies *multiplex* and *fastidiosa* have been detected in *P. spumarius* (Cesbron et al., 2020; Moralejo et al., 2020), and under experimental conditions, their role as a vector has been confirmed for bacterial transmission between almond and almond, vine to vine, and vine to almond tree (Cesbron et al., 2020; Moralejo et al., 2020). Other insects considered potential vectors in Majorca are *N. campestris* and *N. lineatus* (Olmo et al., 2021). Morente et al. (2018) showed that in Alicante, *P. spumarius* and *N. campestris* are frequently found on weeds in olive groves. However, there are no data on vector insects in almond orchards affected by ALSD. In France, *P. spumarius*is is considered the main vector for both diseases in Corsica; and in the region of PACA, captured specimens showed both strain types (ST6 and ST7) of subsp. *multiplex* present in these regions (Cunty et al., 2020).

## Conclusions

Cultivated species of *Prunus* are grown all over the world and some are a significant source of income for thousands of growers. In addition to the economic value that crops such as almonds, plums, and peaches have in some countries, the landscape value of these plants is also significant for historical reasons and traditions that strongly link these species to the culture of the local population.

ALSD is the most critical almond disease in the United States, and together with PD in grapes, many studies have been conducted on this disease. ALSD has been detected in several parts of Europe and Asia; and in some parts of these areas, the damage seems to be severe, similar to that experienced in Spain and Iran. Conversely, in other areas such as Italy, the impact is less, probably because of different cultivars or subspecies.

The bibliographic analysis shows Europe has recently been the center of research on *X. fastidiosa* and almond trees. From 2013 to 2020, 36.4% of the total number of studies published on this topic/host were related to European research. However, these studies mostly focused on the spread of the disease (ALSD) (33.3% of studies), sequence typing of isolates (16.6%), and vectors involved in the epidemics (16.6%). In contrast to the studies conducted in North America, in Europe and Asia, no resistant or tolerant almond cultivars have yet been identified, either in the field or after cross-breeding and selection processes, and few studies have been carried out on the issue of resistance in stone fruits. Furthermore, studies on the resistance conferred by rootstocks have not yet been carried out in Europe. All this is justified by the fact that the European outbreaks of X. *fastidiosa* are recent.

PLS and PPD are a well-known threat to the agricultural sector of the United States and South America, and for decades, field observations and research have contributed to understanding the epidemiology and the role of the vector in the spread of these diseases. In most European and Asian areas, these diseases are not yet present, or they have not yet been detected; however, in the unfortunate hypothesis of pathogen introduction, they could compromise entire production sectors.

China and some countries of the Balkan peninsula (Romania, Serbia, and Montenegro) are important centers of plum production; and in China, Italy, Spain, and Greece, peach growing is one of the most economically important types of cultivation (www.fao.org). Among the species of the genus *Prunus*, new varieties tolerant to PLS, both in Brazil and Florida, have only been successfully obtained for the Japanese plum. In Brazil, it is hypothesized that the tolerance is due to the vector tending to feed on other cultivars, although further confirmation is necessary. The analysis also indicates that where *X. fastidiosa* causes diseases in stone fruits, few studies have yet been carried out on the defense mechanisms implemented by the plant to counteract the development of the pathogen (resistance) or to reduce its symptomatic effects (tolerance). Only 10.93% of the studies explored the theme of tolerance, resistance, and cultivar selection (10.15% from the Americas and.78% from Europe), and 42.86% of these studies involved plums. Furthermore, in many of the studies on varietal response to infection, those assessing the status “resistant” or “tolerant” are not always sufficiently comprehensive.

The analysis revealed that there is little research on potential European vectors of *X. fastidiosa* in species of the genus *Prunus* despite the fact that confirming the vector(s) of the pathogen means that containment strategies can be set up in time.

Considering the lines of research developed to date and the unavailability of effective treatments, research, therefore, needs to focus on resistance mechanisms in *Prunus* species and on the interactions between the plant and pathogen. It is also important to characterize the entomofauna of countries where *X. fastidiosa* is not yet present, in order to identify potential vectors, and, carry out transmission tests to confirm the role of insects in the spread of the disease, especially in countries where it is present,

In Europe, the selection of resistant or tolerant almond cultivars in the field is urgently needed. In addition, crossing and selection activities are needed to give almond growers a wider biodiversity and the possibility of recovering the areas affected by ALSD, which is probably the most impacting disease caused by *X. Fastidiosa* in stone fruit.

## Author Contributions

DG and AA wrote the manuscript and contributing major parts of the literature survey. LD and AL wrote the article and reviewed the manuscript. AL provided research supervision. All the authors collaborated in the writing process, read, and agreed with the published version of the manuscript.

## Conflict of Interest

The authors declare that the research was conducted in the absence of any commercial or financial relationships that could be construed as a potential conflict of interest.

## Publisher's Note

All claims expressed in this article are solely those of the authors and do not necessarily represent those of their affiliated organizations, or those of the publisher, the editors and the reviewers. Any product that may be evaluated in this article, or claim that may be made by its manufacturer, is not guaranteed or endorsed by the publisher.

## Footnotes

^1^PLS was observed in 31 *P. salicina* cultivars: Amarelinha, America, Blood, Burbank, Carmesin, Chabot, Cherry, The Firts, Friar, Georgetown, Golden Japan, Hale, Kahinta, Kelsey Paulista, Methley, Ozark Premier, Pluma 2, Pluma 3, Pluma 4, Pluma 5, Pluma 6, Pluma 7, Rosa Grande, Roxa de Itaquiera, Santa Rita, Santa Rosa, Sanguinea, Satsuma, Sugar Jaboticaba, Ura Boni, and Wickson; and in six *P. domestica* cultivars: Anna Spath, Reine Claude, d'Agen, Imperial, President, and Tragedy (French and Feliciano, 1982).
